# Significance of coagulase negative *Staphylococcus* from blood cultures: persisting problems and partial progress in resource constrained settings

**Published:** 2016-12

**Authors:** Shailpreet K. Sidhu, Sita Malhotra, Pushpa Devi, Arpandeep K. Tuli

**Affiliations:** Department of Microbiology, Govt Medical College, Amritsar, Punjab, India

**Keywords:** Coagulase negative *Staphylococcus*, Bloodstream infections, Contaminants

## Abstract

**Background and Objectives:**

Coagulase negative *Staphylococcus* (CoNS) is frequently isolated from blood cultures but their significance is difficult to interpret. CoNS bacteria which are often previously dismissed as culture contaminants are attracting greater importance as true pathogens in the past decades. Clinical evaluation of these isolates suggests that although there is a relative increase of CoNS associated bloodstream infections in recent years, the microorganisms still remain the most common contaminants in blood cultures. The objective of this study was to determine the significance of CoNS isolated from blood cultures.

**Materials and Methods:**

A retrospective study was conducted to evaluate the rate of contamination in blood cultures in a tertiary care hospital. The paired specimens of blood were cultured using conventional culture methods and the isolates of coagulase negative staphylococci were identified by standard methodology. Clinical data, laboratory indices, microbiological parameters and patient characteristics were analyzed.

**Results:**

Of 3503 blood samples, CoNS were isolated from blood culture of 307 patients (8.76%). The isolates were reported as true pathogens of bloodstream infections in only 74 out of 307 cases (24.1%). In the vast majority, 212 of 307 (69.0%), they were mere blood culture contaminants and reported as insignificant/contaminant.

**Conclusion:**

Determining whether a growth in the blood culture is a pathogen or a contaminant is a critical issue and multiple parameters have to be considered before arriving at a conclusion. Ideally, the molecular approach is for the most part a consistent method in determining the significant isolates of CoNS. However, in countries with inadequate resources, species identification and antibiogram tests are recommended when determining significance of these isolates.

## INTRODUCTION

Although it has been widely appreciated by physicians and microbiologists that blood cultures are among the most important laboratory tests performed in the diagnosis of serious infections, it has become equally apparent in more recent years that contaminated blood cultures are common, enormously costly and frequently confusing for clinicians (1). Clinical studies of bloodstream infections over 3 decades have provided guidelines for differentiating true pathogens from contaminants or microorganisms of unknown significance. However a true “gold standard” for differentiating pathogens from contaminants does not exist (2). Moreover, the most common blood culture contaminants, coagulase-negative staphylococci (CoNS) have been proven to be especially problematic (3).

There is no doubt that coagulase negative staphylococci are the most common isolates from blood cultures. But are they the most common pathogens as it has been reported? If not, how often are these CoNS isolates the true pathogens of bloodstream infections and how often are they mere blood culture contaminants? These are the questions for which there is no clear-cut answer and the points which continue to be debated (4).

The infections which are caused by these microorganisms, CoNS, are increasing as the number of catheters and artificial devices which are being inserted through the skin becomes higher (5). Clinical evaluation of these isolates suggest that although there is a relative increase of CoNS associated bloodstream infections in recent years, these microorganisms still remain the most common contaminants in blood cultures. Clinical criteria in predicting whether isolated CoNS from blood cultures are associated with bloodstream infection are neither sensitive nor specific (6). In order to be clinically significant, repeated CoNS should belong to the same strain and should be confirmed by genotyping methods which are not widely available. These uncertainties regarding the significance of CoNS isolated from blood cultures may result in over-diagnosis and indirectly overuse of anti-staphylococci drugs especially vancomycin which may contribute to the development of resistance that will amplify the likelihood of morbidity, mortality and total hospital costs (7). Thus, the objective of this study was to determine the significance of CoNS isolated from blood cultures.

## MATERIALS AND METHODS

### Sampling and culture conditions

A retrospective study was conducted from July 2014 to December 2015 in the Department of Microbiology, Government Medical College and Hospital, Amritsar to evaluate the rate of contamination in the blood cultures in a tertiary care hospital. A total of 3503 blood samples were tested by culture method. Two sets of blood cultures were taken from every patient using strict aseptic precautions and inoculated immediately in BHI broth and were plated on 10% sheep blood agar and MacConkey agar plates after 24 and 48 hours of incubation. The negative result was followed-up by examining the broth daily and doing a final subculture at the day 7. These plates were incubated for 18–24 hrs at 37°C and the growth of coagulase negative staphylococci was tested by standard methodology (8) including Gram staining, catalase and coagulase test. Antimicrobial sensitivity was determined by Kirby Bauer disc diffusion method as per CLSI guidelines (9).

Clinical data, laboratory indices, microbiological parameters and characteristics of patients who had pure growth of CoNS in their blood cultures were analyzed. Data were collected using a designed protocol similar to that used by other authors in studies on bloodstream infections (10, 11).

Essential clinical criteria for true bacteremia included one or more of the following factors (12): Persistent fever ≥ 38°C or body temperature below 36°C, hypotension (BP< 90 mmHg), neutropenia with left shift differential or disseminated intravascular coagulopathy (DlC). Other risk/predisposing factors such as intravenous catheter or indwelling foreign devices, immunosuppressed patients with underlying illnesses (peritoneal dialysis or hemodialysis patients, postsurgical infections), stay in intensive care unit, antimicrobial therapy including duration and clinical response to the therapy, duration of hospitalization and any other evidence of laboratory infection were also included.

Laboratory criteria for classification of true bacteremia included patients with the same isolated bacteria from at least two sets of blood cultures or patients with the same isolated species in one set of the initial blood samples, incubation time for bacterial growth (duration of obtaining positive blood culture results), date of blood sampling and result of culture from any other site. Patients who were receiving antibiotics prior or during blood sampling were excluded from this study.

## RESULTS

A total number of 3503 blood samples were received over a period of one and a half years. Coagulase negative staphylococci (CoNS) were isolated from blood culture of 307 patients (8.76%). The majority of patients (n=188) were adults in the age range of 31–75 years whereas 119 were pediatric (0–12 yrs) with CoNS positive blood cultures. Frequency of CoNS isolates was higher in pediatric department (38.7%), followed by intensive care units (31.3%), medical wards (18.1%), surgical wards (8.5%) and obstetrics department (3.4%). All positive blood cultures were further evaluated to ascertain the clinical significance of CoNS. Time duration to become CoNS positive showed that among the total 307 samples, 109 grew within 48 hours after incubation, 165 during 72 hours and only 33 samples grew beyond 72 hrs. The isolates were reported as true pathogens of bloodstream infections in 74/307 (24.1%) samples. The most frequently observed category was assumed as blood culture contaminants (n=212; 69.0%) which were reported as insignificant/contaminant isolates. Clinical significance could not be accurately ascertained in 21 isolates (6.9%) which were reported as undetermined and suggested to be rechecked by culture test.

Among 74 clinical significant isolates, 63 isolates (85.1%) grew within 48 hrs. Risk factors and other associated diseases were also studied among this group of patients (Table 1).

Table 2 demonstrated antibiotic therapy of 212 patients who represented contaminating coagulase negative staphylococci in their blood cultures. Antibiotics were not prescribed for 29 patients (13.6%) probably due to the recognition of contaminating isolates by the clinicians. Approximately 80% of the patients in this group received anti-staphylococcal antibiotics.

**Table 1.** Criteria/Risk factors among patients infected with significant CoNS (n=74)

**Criteria/Risk Factor**	**No. (%)**
Body temperature (> 38°C or < 36°C)	65 (87.8)
TLC (> 12000 or < 4000)	55 (74.3)
Hypotension (Systolic BP< 90)	29 (39.2)
Sepsis	57 (77.0)
Intravascular catheters	53 (71.6)
Invasive devices/procedures	21 (28.3)
Underlying immunosuppression	49 (66.2)
Duration of hospitalization (> 48 hrs)	68 (91.9)
Staying in ICU	27 (36.5)
Time for positive culture test:	
• ≤ 48 hrs	63 (85.1)
• >48 hrs	11 (14.8)

**Table 2.** Antibiotic therapy in 212 patients showed contaminating CoNS

**Antibiotic Therapy**	**No. of Patients (%)**
No Antibiotic	29 (13.6)
Vancomycin	27 (12.7)
Linezolid	61 (28.9)
Other anti-staphylococcal antibiotics	95 (44.8)

## DISCUSSION

Blood sampling for culture test is a routine procedure to investigate the cause of fever or suspected infection in the majority of hospitalized patients. Isolation of a true pathogen from blood culture ultimately warrants treatment with an appropriate antibiotic. Problems occur when the isolated microorganism is of doubtful significance such as CoNS which require further clinical assessment and extra laboratory tests to help the physician in appropriate patient management. Coagulase-negative staphylococci (CoNS), the most frequent blood culture isolates, are predominantly blood culture contaminants but they are also significant causes of bacteremia (11, 13). For clinical microbiologists, interpretation of the clinical significance of isolated CoNS from blood culture continues to be complicated. Blood isolation of CoNS in more than one occasion and determination of various clinical parameters are usually used to detect the clinical significance of the isolates.

Clinical and microbiological guidelines have been published for differentiation of true bacteremia from pseudobacteremia or contamination (12, 14). Suggested laboratory criteria for true bacteremia include growth within 48 hrs and multiple blood cultures for the same microorganism. In contrast, increased growth time before getting positive culture result, multiple growth of skin microorganisms or bacterial growth during antibiotic treatment can be assumed as contamination. It also has been recommended that addition of clinical guidelines is essential for appropriate classification of bacteremia (11). Furthermore, due to their low virulence, they may not evoke sufficient inflammatory responses and a group of patients with CoNS bloodstream infections may not have typical clinical manifestations and laboratory indices of the infection. In addition to the standard criteria for infections, other certain criteria have to be taken into account to ascertain the significance of the isolates which include: (i) Number of taken blood samples: In the present study, paired blood samples were obtained from all patients. It has been defined that isolation of same CoNS strains from multiple blood samples taken from different body sites or at different time intervals is more likely to be a pathogen than a contaminant (15) but it is not an absolute indicator of the infection and interpretation of clinical significance should not be based solely on this criterion. It should be used together with other parameters in ascertaining the significance (16). (ii) Clinical condition of the patient: CoNS rarely ever causes bloodstream infection in a healthy immunocompetent young adult without any predisposing factor. Hence a predisposing factor such as central venous catheter, indwelling foreign devices, age of the cases (especially premature neonates), immunocompromised and undernourished states, a ventriculovenous shunt or any other predisposing factors should be looked for and correlated in the evaluation (17). We studied various risk factors and other associated diseases and found that 24.1% of CoNS positive cases had underlying predisposing diseases. (iii) The growth time: time duration for obtaining positive culture test showed that among 307 samples, 109 CoNS grew within 48 hrs, 165 isolates in 72 hrs and only 33 isolates grew beyond 72 hrs. Generally, pathogenic bacteria originated from blood samples grow in a shorter time than contaminants (18). (iv) Quantity of the bacterial growth: a low colony count should not be dismissed as a contamination in a high risk population (19). (v) Source of sampling: when positive blood samples are taken from catheters three possibilities should be considered: True bacteraemia, catheter colonization or contamination. The catheter colonization may or may not progress to cause symptoms of infection or true bacteraemia. 15–25% of short-term central venous catheters are usually colonized by CoNS and most of them have no evidence of infection. Thus, a substantial number of patients who have central venous catheters (CVC) are expected to be positive due to the catheter colonization (20).

In our laboratory, a protocol is followed to determine the significance of CoNS bacteremia based on mentioned criteria (10) including clinical parameters, sepsis, isolation of CoNS from blood on more than one occasion, presence of underlying diseases or immunosuppression. (a) If two or more blood samples are submitted and only one becomes positive, neither species identification nor susceptibility testing will be done and the isolate will be reported as a probable contaminant. (b) If only one blood sample is submitted (especially in pediatrics) and it represents CoNS, clinical parameters and underlying conditions (as mentioned before) will be assessed. If no sign or symptom of sepsis or no underlying risk factor is present, the isolate is reported as undetermined significance or probable contaminant. In the cases of any sign/symptoms of sepsis or any other underlying risk factor in the absence of any other obvious source of infection, identification and susceptibility results will be reported. (c) If two or more blood samples represent CoNS, a full workup will be done. If the isolated strains show the same biochemical profiles and antibiograms, it is probable that they are identical and are therefore significant and will be reported. However, if the biochemical profiles and antibiograms are different, the isolates will be much more likely to represent contaminations. Furthermore, it is important for clinicians to mention the detailed clinical data and relevant history of the patient in the request form so that microbiologist can correlate culture results with the clinical findings before final identification and susceptibility reports.

In our study, after considering both clinical and laboratory parameters, CoNS were considered true pathogens of blood stream infections in only 24.1% of the cases. In vast majority of the cases (69.0%), they were reported as contaminants. Similar study was conducted by Bodonaik and Moonah (11) in which 73.3% of the isolated CoNS were reported as blood culture contaminants. Other studies also reported pseudobacteremia due to the CoNS isolates as mere blood culture contaminants (12, 21). In another study, CoNS were accounted for 45 to 60% of total blood isolates among which 10–12% were implicated in significant bloodstream infections (13).

In the present study, nearly 80% of the patients showed contaminating CoNS received anti-staphylococcal antibiotics. There is a tendency amongst clinicians and clinical microbiologists to overuse antibiotics in patients suffered from coagulase negative staphylococci in their blood samples. One-third of our studied patients showing contaminating CoNS were treated with specific anti-staphylococcal antibiotics which has reached to 50% in the study conducted by Souvenir et al. (13).

This data clearly illustrates the difficulties in ascertaining the clinical importance of CoNS isolates from blood samples in routine laboratories. Despite detailed evaluation, the authors were unable to accurately ascertain the clinical significance in nearly one fifth of the patients which has been confirmed by other studies (12, 13, 22). Various clinical criteria should be considered in prediction of the importance of CoNS isolates from blood samples. Repeatedly isolated CoNS strains should be from the same origin in order to be clinically significant and should be confirmed by genotyping methods which are not widely available (23). Many laboratories in developing countries cannot afford doing routine strain typing. Comparison of antibiotic susceptibility pattern (antibiotype) is an alternative way in such situations.

## CONCLUSION

Coagulase negative staphylococci from blood samples were occasionally dismissed as mere contaminants. Recently, in regard to the changes in therapeutic modalities, there has been an increased rate of coagulase negative staphylococcal bloodstream infections. This should be kept in mind that patients suffered from coagulase negative staphylococci isolates in their blood samples should be carefully evaluated before instituting therapy to avoid unnecessary use of antibiotics and the consequent increase of antibiotic resistance patterns in hospitals. Ideally, molecular approaches are consistent methods to determine significant CoNS isolates. However, in countries with inadequate resources, proper clinical data, multiple blood sampling and antibiogram testing are recommended to assess significance of CoNS*.*
